# Encouraging visual outcomes in children with idiopathic and JIA associated uveitis: a population-based study

**DOI:** 10.1186/s12969-023-00841-8

**Published:** 2023-06-15

**Authors:** Mira Siiskonen, Iida Hirn, Roosa Pesälä, Pasi Ohtonen, Nina Hautala

**Affiliations:** 1 Department of Ophthalmology, Oulu University Hospital, Oulu, Finland; 2 Research Unit of Clinical Medicine, Oulu, Finland; 3 Medical Research Center, University of Oulu, Oulu, Finland; 4 The Research Unit of Surgery, Anesthesia and Intensive care, Oulu University Hospital and University of Oulu, Oulu, Finland; 5 Research Service Unit, Oulu University Hospital, Oulu, Finland; 6 Research Unit of Clinical Medicine and MRC Oulu, Department of Ophthalmology, University of Oulu, Oulu University Hospital, Oulu, P.O.Box 21, 90029 OYS Finland

**Keywords:** Uveitis, Idiopathic, JIA-U, Visual acuity, Visual outcome

## Abstract

**Background:**

Pediatric uveitis is typically asymptomatic and may become chronic affecting ocular structures and vision. We evaluated visual outcomes, clinical features, medication, and uveitis activity in children with either idiopathic uveitis (idio-U) or juvenile idiopathic arthritis associated uveitis (JIA-U).

**Methods:**

A longitudinal, population-based cohort study of children with uveitis in 2008–2017. The data included parameters for age, gender, age at diagnosis, laterality, chronicity, anatomical distribution, etiology, systemic association, uveitis activity, medication, and visual outcomes.

**Results:**

A total of 119 patients aged < 16 years with uveitis were included. Uveitis was idio-U in 23% and associated with JIA in 77% of cases. 37% of the patients in the idio-U group and 65% in the JIA-U were girls (p = 0.014). The mean age at first uveitis was 10.0 (SD 3.4) years in idio-U and 5.5 (SD 3.3) years in JIA-U (p < 0.001). Anterior location of uveitis was noted in 74% in idio-U and 99% in JIA-U (p < 0.001). Mostly, uveitis was chronic (59% in idio-U and 75% in JIA-U) and bilateral (56% in idio-U and 64% in JIA-U). Topical corticosteroids were initially used by 89% and 100%, systemic corticosteroids by 30% and 27% in some extent during the follow-up, disease-modifying antirheumatic drugs (DMARDs) by 33% and 85% (p < 0.001) of the patients in idio-U and JIA-U, respectively. Biologic disease-modifying antirheumatic drugs (bDMARDs) were more common in JIA-U (55% vs. 15% in idio-U, respectively, p < 0.001). Most patients had normal visual acuity (Snellen > 0.8, [6/7.5]) in the affected eye and bilaterally in 85% idio-U and 70% JIA-U. Only 5 patients (4%) had visual impairment in one, but none in both eyes. Uveitis activity by SUN classification was 0 + in 81% and 72%, 0.5 + in 19% and 25%, and 1 + in 0% and 3% in the idio-U and JIA-U, respectively.

**Conclusions:**

Children with uveitis have good visual acuity and a low rate for visual impairment. In addition, modern treatment with DMARDs and bDMARDs seems to save vision.

## Background

Pediatric uveitis is rare and typically asymptomatic, which may lead to diagnostic challenges and delay of the treatment despite the severe inflammation, especially in cases of non-infectious and chronic uveitis [1, 2]. Uveitis in children accounts for only 2–20% of all uveitis worldwide [3]. However, the incidence of pediatric uveitis in Finland has been shown to increase from 4/100 000 in 2000 to 14/100 000 in 2008–2017 [4–6]. Furthermore, the prevalence of pediatric uveitis doubled during the past two decades possibly due to rising prevalence of juvenile idiopathic arthritis (JIA) in children [4–9]. The developments in monitoring and diagnosing uveitis in pediatric patients with JIA have developed during the last decade, which may at least partly explain the increased rates of pediatric uveitis [10]. Uveitis in children is commonly chronic, and the risk of developing ocular complications is high [3, 11]. Inflammation may cause damage to ocular structures and gradually affect vision [12, 13]. Modern treatment modalities, however, allow effective treatment of pediatric uveitis, which is often resistant to conventional treatment and causes ocular complications [3, 14].

Most pediatric uveitis is idiopathic (idio-U) or associated with JIA (JIA-U) [1, 2, 15]. The known risk factors for the development of JIA-U in pediatric patients are oligoarticular pattern of arthritis, age under 7 years at onset of arthritis and antinuclear antibody positivity [16, 17]. Previous studies have shown that 50–75% of the children with severe uveitis are at risk for developing visual impairment due to ocular uveitis-related complications [11, 14, 18]. However, the knowledge of the long-term effect of pediatric uveitis, uveitis activity and medication on visual outcomes of pediatric patients with uveitis is limited.

Purpose of this study is to evaluate long-term visual outcomes in a population-based cohort of children with either idio-U or JIA-U [4]. In addition, the effect of modern medication on uveitis activity and vision is compared between the groups.

## Methods

This retrospective interventional case series was performed at Oulu University Hospital. The study followed the tenets of the Declaration of Helsinki and it was conducted with the approval of the Oulu University Hospital Research Committee (89/2017).

The population-based cohort consisted of all children with uveitis in Northern Ostrobothnia Hospital District during the study period in 2008–2020. The treatment of pediatric uveitis was completed at Oulu University Hospital responsible for tertiary care for a population of approximately 410 000 inhabitants. All pediatric patients < 16 years of age who presented at Oulu University Hospital with idio-U and JIA-U between Jan 1, 2008 and Dec 31, 2017, were included. The hospital’s electronic patient database was used to search for the patients with uveitis by using the ICD-10 (International Classification of Diseases) diagnose codes for anterior (H20.0 and H20.1), intermediate (H30.2) or posterior uveitis (H30.0-H30.9). Patients with uveitis other than idio-U or JIA-U were excluded. Demographic data was collected and included parameters for age, gender, age during the first episode, laterality, chronicity, anatomical distribution of the disease, etiology (idio-U or JIA-U), best-corrected visual acuity (BCVA), and treatment (topical or systemic corticosteroids, dexamethasone implant, disease-modifying antirheumatic drugs (DMARDs), biologic therapy (bDMARDs)). Classification of the anatomical location and chronicity of uveitis was based on the Standardization of Uveitis Nomenclature (SUN) classification [19]. JIA was diagnosed in children according to the International League of Associations for Rheumatology Classification [20] and screening for JIA-U was carried out according to the screening guidelines described earlier by Heiligenhaus et al. [10]. Visual acuity was evaluated by the Snellen chart, and the affected eye or the eye with more uveitis activity and/or worse BCVA in cases of bilateral uveitis was analyzed. The WHO criteria of distance vision impairment (Snellen visual acuity < 0.3 [6/18] or < 59 ETDRS letters in a better eye) was used. For the treatment of uveitis in children, methotrexate was started if there was a need for the topical corticosteroids over 2 drops per day after one month of treatment. If there was no treatment response to methotrexate in three months, or there was ocular hypertension (intraocular pressure > 25 mmHg) or other complications of uveitis (poor visual acuity, cataract, macular edema or papilledema or epiretinal membranes), adalimumab was added to the treatment [21–24]. Dexamethasone implant was inserted for diagnosed macular edema or to prevent postoperative inflammation after cataract surgery in the high-risk patients included in the present cohort.

Summary data are presented as mean with standard deviation (SD) for the continuous variables. Categorical data are expressed as frequencies with percentages. The Student’s t-test was used for between group comparisons for continuous, normally distributed data and Mann-Whitney U test for not normally distributed data. The chi-square test or the Fisher’s test was used to evaluate the differences between categorical data.

Statistical significance was set at p value < 0.05. The SPSS for Windows (IBM Corp. Released 2021. IBM SPSS Statistics for Windows, Version 28.0. Armonk, NY: IBM Corp) was used to perform all statistical evaluations.

## Results

The characteristics of the study population are presented in Table 1. A total of 119 patients aged < 16 years with uveitis were included, out of whom 27 (23%) had idio-U and 92 (77%) had JIA-U. The mean follow-up time was 97 (SD 57) months (73土55 months for idio-U and 104土56 months for JIA-U). Ten (37%) of the patients in the idio-U group, and 60 (65%) in the JIA-U, were girls (p = 0.014). The mean age at first uveitis was 10.0 (SD 3.4) years in idio-U, and 5.5 (SD 3.3) years in JIA-U (p < 0.001). Anterior location of uveitis was noted in 20 (74%) patients in idio-U and 91 (99%) patients in JIA-U (p < 0.001). In most patients uveitis was chronic (59% in idio-U and 75% in JIA-U) and bilateral (56% in idio-U and 64% in JIA-U). Increased Ana-Ab (≥ 320) were noted in 9% and 45% in idio-U and JIA-U, respectively (p < 0.001).

**Table 1:** Demographic and clinical features of the study cohort of children with either idiopathic uveitis (idio-U) or juvenile idiopathic arthritis associated uveitis (JIA-U)

	idio-U	JIA-U	p-value
	n = 27 (23%)	n = 92 (77%)	
Girls, n (%)	10 (37)	60 (65)	0.014
Mean age at first uveitis, years (SD)	10.0 (3)	5.5 (3)	< 0.001
[Median]	[10]	[4]	
Follow-up time (months), mean (SD)	73 (55)	104 (56)	0.011
[Median]	[59]	[100]	
Uveitis location, n (%)			< 0.001
Anterior	20 (74)	91 (99)	
Intermediate	6 (22)	1 (1)	
Posterior	1(4)	0 (0)	
Chronicity, n (%)			0.143
Acute	6 (22)	8 (9)	
Recurrent	5 (19)	15 (16)	
Chronic	16 (59)	69 (75)	
Laterality, n (%)			0.500
Bilateral	15 (56)	59 (64)	
Ana-Ab ≥ 320, n (%)	2 (9)	41 (45)	< 0.001
Missing, n (%)	4 (15)	1 (1)	

Most study patients used topical corticosteroids at the onset of uveitis (89% in idio-U and 100% in JIA-U, p = 0.036). Systemic corticosteroids were used by 30% and 27% in idio-U and JIA-U, respectively. DMARDs were used in 33% in idio-U and 85% in JIA-U (p < 0.001). bDMARDs were more commonly used in the JIA-U group than in patients with idio-U (55% vs. 15%, respectively, p < 0.001) (Table 2).

**Table 2:** Treatment used by the study patients with idiopathic uveitis (idio-U) or juvenile idiopathic arthritis related uveitis (JIA-U). The patients were commonly using several medications simultaneously depending on the severity of ocular inflammation. Only one bDMARDs, however, was used at a time

Treatment, n (%)	idio-U	JIA-U	p-value
	n = 27	n = 92	
Topical corticosteroids	24 (89)	92 (100)	0.036
Systemic corticosteroids	8 (30)	25 (27)	0.810
Dexamethasone implant	0 (0)	4 (3)	
DMARDs	9 (33)	79 (85)	< 0.001
bDMARDs	4 (15)	51 (55)	< 0.001
Adalimumab	4 (15)	44 (48)	
Infliximab	0 (0)	9 (8)	
Others	0 (0)	9 (8)	

DMARD; disease-modifying antirheumatic drug

bDMARDs; biologic disease-modifying antirheumatic drug

Others; etanercept (for arthritis), golimumab, tocilizumab, abatacept

A majority, 84%, of all pediatric patients with uveitis had normal bilateral visual acuity (> 0.8 Snellen chart [6/7.5]), 70% had BCVA > 0.8 [6/7.5] in the worse eye, and 92% had BCVA > 0.5 [6/12] in the worse eye (Table 3). Visual acuity of < 0.5 [6/12] in the worse eye was noted in 1 (4%) patient in the idio-U group and 8 (9%) patients in the JIA-U group. Only 5 patients (4%) had visual impairment in one eye, but none had BCVA < 0.3 [6/18] in both eyes.

At the end of 73–104 months follow-up uveitis activity was 0 + in 81% and 72%, 0.5 + in 19% and 25%, and 1 + in 0% and 3% in the idio-U and JIA-U according to SUN classification (Table 3). All three patients (3%) with uveitis activity of 1 + were in the JIA-U group and treated with only DMARDs or bDMARDs. The patients without systemic treatment had lowest uveitis activity (100% had no cells). Those using systemic treatment had uveitis activity grade 0 + in 74%, and + 0.5 in 24% of the cases.

**Table 3:** Visual outcome and uveitis activity at the end of follow-up in different therapy groups in patients with idiopathic uveitis (idio-U) or juvenile idiopathic arthritis associated uveitis (JIA-U). The affected eye or the more active eye with lower visual acuity in bilateral uveitis was analyzed. The numbers represent the number of patients (%) in each treatment category

		No DMARD	DMARD	DMARD + Biologics	p-value
**idio-U**	**Visual outcome, Snellen, worse eye**				0.642
	**<0.5**	1 (6)	0 (0)	0 (0)	
	**0.5–0.8**	3 (18)	1 (17)	2 (50)	
	**>0.8**	13 (77)	5 (83)	2 (50)	
	**SUN classification, n (%)**				0.003
	**0+**	17 (100)	3 (50)	2 (50)	
	**0.5+**	0 (0)	3 (50)	2 (50)	
	**1+**	0 (0)	0 (0)	0 (0)	
**JIA-U**	**Visual outcome, Snellen, worse eye**				0.008
	**<0.5**	0 (0)	1 (3)	7 (14)	
	**0.5–0.8**	2 (17)	2 (7)	17 (33)	
	**>0.8**	10 (83)	26 (90)	27 (53)	
	**SUN classification, n (%)**				0.088
	**0+**	12 (100)	22 (76)	32 (63)	
	**0.5+**	0 (0)	6 (21)	17 (33)	
	**1+**	0 (0)	1 (3)	2 (4)	
**Total**	**Visual outcome, Snellen, worse eye**				0.004
	**<0.5**	1 (3)	1 (3)	7 (13)	
	**0.5–0.8**	5 (17)	3 (9)	19 (35)	
	**>0.8**	23 (79)	31 (89)	29 (53)	
	**SUN classification, n (%)**				< 0.001
	**0+**	29 (100)	25 (71)	34 (62)	
	**0.5+**	0 (0)	9 (26)	19 (35)	
	**1+**	0 (0)	1 (3)	2 (4)	

IU, idiopathic; JIA, juvenile idiopathic arthritis; DMARD, disease-modifying antirheumatic drug

## Discussion

Pediatric uveitis is a rare and challenging disorder, which may lead to visual impairment in its severe forms with ocular complications [3]. Delightfully, modern pharmacological inventions have improved the treatment of uveitis in children [25, 26]. In the present study, we evaluated the clinical characteristics, long-term visual outcomes, the rate of visual impairment, uveitis medication, and uveitis activity in pediatric patients with idio-U or JIA-U.

Regular monitoring of children with JIA may also explain the young age, 5.5 years, at the diagnosis of uveitis in the present cohort compared to the median age of 9.4 years at diagnosis of pediatric uveitis reported previously [27]. In agreement with previous study, uveitis was more common in girls in both idio-U and JIA-U [2]. Most uveitis in children are non-infectious and idiopathic, and JIA is the most common systemic disease associated with the pediatric uveitis [1, 4, 6, 10, 15]. In agreement with these previous studies reporting JIA as an identified cause of pediatric anterior uveitis in 50–80% of cases, 61% of all cases of pediatric uveitis in the area [4], and 77% of the patients in the present study had JIA-U possibly due to rising prevalence of JIA in children [5, 6, 28]. Epidemiological studies have demonstrated that the Finnish population is notably prevalent with JIA and the disparity in the incidence and prevalence of JIA depending on geographical location, ethnicity, classification of arthritis and time of the studies [29–32]. Recent study from the United States, for example, reported that JIA caused anterior uveitis in 48% of the cases in a cohort of 134 pediatric patients [33]. Positive antinuclear antibodies, young age and female sex are known risk factors for developing chronic and bilateral uveitis, which was the case in 75% and 64% of the study patients with JIA-U. Uveitis location was anterior in 74% of the cases with idio-U and 99% in the JIA-U in the present cohort. Anterior uveitis has been reported to be the most common form of uveitis in 44–52% of all pediatric patients [2, 27, 34], and a higher, 62%, prevalence of anterior location of uveitis was reported recently from the United States [35]. The increasing trend in higher prevalence of JIA-U and the decreased proportion of posterior inflammatory uveitis might explain this change and a high number of anterior uveitis in our study [1, 4, 6, 10, 15].

Topical corticosteroids are the first-line treatment for pediatric anterior uveitis and were used by 89% and 100% of the patients in idio-U and JIA-U groups in the present study. One third of all patients with childhood uveitis used systemic steroids during the follow-up period, and 4 patients in the JIA group had received intravitreal dexamethasone implant. DMARDs and bDMARDs (corticosteroids sparing therapies) were used in 46% of the participants, more commonly in patients with JIA-U than idio-U. The use of bDMARDs in our cohort seems to be in line with the previous studies reporting that biologics were used in 35–49% of the pediatric patients with uveitis [33, 36, 37]. The use of bDMARDs has been shown to greatly improve the outcome of non-infectious uveitis both in pediatric and adult patients with uveitis. The recent SYCAMORE and ADJUVITE trials have proven the efficacy and safety of adalimumab in children with JIA-U [38, 39]. In agreement, only 4% (2/55) of patients using bDMARDs in the current cohort had uveitis activity of 1 + at the end of follow-up supporting the effectiveness of the modern treatment on uveitis activity. Especially in pediatric uveitis, early and efficient treatment of ocular inflammation is important for preventing the development of vision-threatening complications, particularly in children at amblyopic age. Consideration of the challenges in compliance to treatment is also needed when choosing the uveitis medication in young children. In further follow-up of patients with pediatric uveitis for the next decade, the use of DMARDs and bDMARDs might be expected to increase. Close collaboration with the pediatric rheumatologists improves the treatment of pediatric uveitis patients.

Uveitis related ocular complications are documented in 25–50% of pediatric patients with uveitis [11, 40, 41]. Decreased visual acuity in up to 40% of patients or visual impairment in one fourth has been reported previously [13, 27, 40]. Pediatric patients with the most severe uveitis are at most risk for ocular complications, and 20–75% of them will gradually develop visual impairment due to complications of uveitis, such as cataract, glaucoma, band keratopathy or macular edema [11, 14, 15]. The rate for uveitis related visual loss seems to have diminished during the last decade, and Markomichelakis et al. recently reported 23% visual loss in pediatric patients with posterior uveitis and 20% in those with panuveitis [15]. However, most of our study patients had anterior uveitis, which is linked to less visual disturbances compared to posterior location of uveitis. In our cohort almost all, 84%, of pediatric patients with either idio-U or JIA-U had normal bilateral visual acuity, and 70% had BCVA > 0.8 and 92% had BCVA > 0.5 in the worse eye. These results agree with the recent studies from Scandinavian countries demonstrating a decrease in BCVA < 0.5 in 0–5% of the patients with JIA-U [37, 42]. Only 5 patients (4%) in our cohort had BCVA < 0.3 in the worse eye, but none had bilateral visual impairment, which is in line with the previous study by Wennink et al. reporting improved visual prognosis of JIA-U in terms of decline in visual impairment from 8 to 0% after 2010 [25]. Most of the participants in the current study had JIA-U, in which vision-threatening ocular complications are rare compared to idio-U, as reported recently [25, 26].

The retrospective nature of the study is a certain limitation. Also, comparison of visual acuity especially in young children at various stages during their normal vision development is challenging. To solve this, we determined visual acuity at the end of the follow-up when vision was comparable to the standard values used commonly in adults. A long follow-up and a fully covered population-based cohort of children with idio-U or JIA-U might be considered as strengths of the study. However, further studies in larger populations are needed to fully elucidate the impact of modern treatment on long-term visual outcomes and the rates for visual impairment due to pediatric uveitis.

## Conclusions

Our results show improvement in visual prognosis of idio-U and JIA-U in children. Early diagnosis of uveitis and efficient treatment with modern medications including DMARDs and bDMARDs are crucial in preventing visual loss. The rate for visual impairment has decreased and no patient in the present study population developed visual impairment.

## Data Availability

The datasets used and/or analyzed during the current study are available from the corresponding author on reasonable request.

## Abbreviations

BCVA: Best-corrected visual acuity
bDMARDs: Biologic disease-modifying antirheumatic drugs
DMARDs: Disease-modifying antirheumatic drugs
ICD-10: International Classification of Diseases
idio-U: Idiopathic uveitis
JIA: Juvenile idiopathic arthritis
JIA-U: Juvenile idiopathic arthritis associated uveitis
SUN: The Standardization of Uveitis Nomenclature classification
